# Hydration and Molecular Dynamics Simulation of Carbonated and Hydrophobically Modified Municipal Solid Waste Incineration Fly Ash Mortars

**DOI:** 10.3390/ma19183994

**Published:** 2026-09-19

**Authors:** Yi Zheng, Kangjie Zhang, Jingwei Zhang

**Affiliations:** 1 School of Civil Engineering, Zhengzhou University, Zhengzhou 450001, China; zhengyi2021@gs.zzu.edu.cn (Y.Z.);; 2 Zhengzhou University Construction Engineering Quality Research and Testing Co., Ltd., Zhengzhou 450002, China; 3 School of Water Conservancy and Transportation, Zhengzhou University, Zhengzhou 450001, China

**Keywords:** municipal solid waste incineration fly ash, carbonation modification, hydrophobic modification, sulfoaluminate cement mortar, molecular dynamics simulation

## Abstract

**Highlights:**

- Carbonation increased strength by 6.8 MPa and reduced water absorption by 17.8%.
- Hydrophobic modification reduced water absorption by 32.5% but lowered strength.
- CWF–C-S-H showed the strongest interfacial binding energy of −2.00 J/m^2^.
- Carbonation is preferred when hydration and mechanical compatibility are prioritized.
- Hydrophobic modification is advantageous for limiting water and ion transport.
- MD results link fly ash modification to C-S-H bonding and chloride mobility.

**Abstract:**

To improve the safe resource utilization of municipal solid waste incineration (MSWI) fly ash in cement-based materials, MSWI fly ash was treated by water washing–carbonation and hydrophobic modification and subsequently incorporated into sulfoaluminate cement mortar. The effects of modified fly ash on hydration reactions, mechanical properties, water absorption, microstructure, and intermolecular interactions of the mortar were systematically investigated. The results showed that water-washed–carbonated fly (CWF) ash promoted early-age hydration, increased hydration heat release, enhanced the compressive strength of mortar by up to 6.8 MPa, and reduced water absorption by up to 17.8%. These improvements may result from the combined effects of desalination during water washing and subsequent carbonation mineralization, in which the CaCO_3_ formed during carbonation can provide heterogeneous nucleation sites and a micro-filling effect, thereby promoting pore-structure refinement. In contrast, hydrophobically modified fly ash retarded cement hydration and reduced the compressive strength by up to 7.7 MPa, but significantly decreased water absorption. Microstructural analysis revealed that CWF promoted the formation of hydration products, including CaCO_3_, C-S-H, and AFt, thereby refining the pore structure. Meanwhile, hydrophobic fly ash reduced the free water content in mortar by introducing hydrophobic groups, weakening continuous capillary water transport and limiting chloride ingress. Molecular dynamics simulations further demonstrated that carbonation modification enhanced the interfacial bonding between fly ash and C-S-H. The CWF–C-S-H system exhibited an interfacial binding energy per unit area of −2.00 J/m^2^, which was higher than those of WF–C-S-H (−1.60 J/m^2^) and HWF–C-S-H (−1.10 J/m^2^), mainly due to enhanced van der Waals and electrostatic interactions. Moreover, MSD analysis indicated that both carbonation and hydrophobic modifications reduced the diffusion capability of mobile species, such as Cl^−^, at the C-S-H interface, providing molecular-scale insights into the stabilization mechanisms of modified fly ash.

## 1. Introduction

Municipal solid waste incineration (MSWI) fly ash is typically enriched with soluble chlorides, alkaline calcium-containing phases, heavy metals, and dioxins. Its environmental risks are governed not only by the total content of contaminants but also by their chemical speciation, mineral composition, pore structure, and environmental pH conditions [1,2,3]. Conventional cement solidification can effectively reduce contaminant migration through precipitation, adsorption, ion exchange, and physical encapsulation. However, under long-term water ingress, acid–alkali fluctuations, or crack development conditions, secondary release of chlorides and heavy metals may still occur [4]. In addition, MSWI fly ash particles generally exhibit loose structures, rough surfaces, and high water absorption capacity. Direct incorporation into cement-based materials may increase water demand and adversely affect hydration reactions, pore connectivity, and durability performance. Therefore, safe resource utilization of MSWI fly ash requires not only reducing the initial leaching risks of contaminants but also achieving a balance among cementitious compatibility, water transport regulation, and long-term environmental stability during service.

Existing pretreatment technologies mainly include water washing, electrochemical separation, mechanochemical activation, cementitious solidification, and high-temperature sintering. Water washing can remove part of the soluble salts and reduce metal emissions during subsequent thermal treatment processes [5]. Electrodialysis facilitates the migration and separation of salts and metal elements through electrochemical driving forces [6]. Mechanochemical treatment can refine particle size, generate surface defects, and enhance the reactivity potential of fly ash as a supplementary cementitious material [7]. Green cementitious materials improve heavy metal immobilization through the formation of hydration products and matrix encapsulation effects [8]. High-temperature sintering achieves detoxification through mineral phase reconstruction and crystalline phase encapsulation [9]. However, water washing inevitably generates high-salinity wastewater, electrochemical treatment involves complex processes and high operational requirements, and thermal treatment suffers from high energy consumption. Meanwhile, conventional cementitious solidification alone remains insufficient to continuously inhibit water-driven migration of contaminants over long-term service periods.

Carbonation treatment utilizes the reaction between reactive calcium phases, such as CaO and Ca(OH)_2_, in MSWI fly ash and CO_2_ to generate stable carbonate phases. This process can reduce the mobility of heavy metals through carbonate precipitation, co-precipitation, surface adsorption, and crystalline encapsulation. High-gravity-enhanced mineralization technology has been employed to achieve the integrated stabilization, solidification, and resource utilization of fly ash [10]. Related reviews further indicate that carbonation degree, residual chloride content, and moisture conditions are critical factors governing CO_2_ sequestration efficiency and heavy metal stabilization performance [11]. Ultrasonic-assisted carbonation can accelerate carbonate formation and improve the mechanical strength and environmental safety of carbonated-fly-ash-based cementitious aggregates [12]. The coupling of mechanochemical activation and CO_2_ mineralization further enhances the reactivity of fly ash as a supplementary cementitious material [13]. In alkali-activated systems, CO_2_ mineralization can also modify precursor dissolution, gel formation, and pore structure evolution [14]. However, existing studies have primarily focused on CO_2_ fixation, heavy metal leaching behavior, and macroscopic strength development, while the hydration kinetics, pore connectivity evolution, and corrosion resistance mechanisms of carbonated fly ash incorporated into sulfoaluminate cement systems remain insufficiently understood.

Hydrophobic modification introduces low-surface-energy functional groups onto particle surfaces or pore walls, thereby reducing material wettability and inhibiting the transport of capillary water and Cl^−^ ions. The incorporation of silane emulsions has been reported to significantly reduce water absorption in concrete, although it may partially inhibit early-age hydration reactions [15]. In recent years, hydrophobic coatings have been widely applied to enhance the water repellency and corrosion resistance of concrete. Their multiscale rough structures and low-surface-energy components can synergistically restrict the ingress of water and aggressive agents [16]. Superhydrophobic coatings also exhibit low permeability and good mechanical stability, demonstrating that regulating interfacial wettability can effectively suppress continuous water transport within concrete [17]. Furthermore, nano-hydrophobic additives can reduce water absorption and chloride ion migration while delaying reinforcement corrosion [18]. Stearate-based integral hydrophobic agents have been shown to decrease water uptake and steel mass loss in cracked mortar [19], while integral superhydrophobic structures also exhibit enhanced corrosion resistance [20,21]. However, hydrophobic modification may alter free water distribution, early-age reaction processes, and microstructural development [22]. In addition, the adsorption of silane molecules on reactive mineral surfaces may hinder the dissolution of hydration products [23].

The alkaline components in MSWI fly ash can react with CO_2_ to generate stable carbonate phases, thereby reducing heavy metal leaching risks and improving the compatibility of fly ash with cement-based systems [24]. However, residual chlorides and porous structures may still compromise the long-term service performance of mortar. Under rainfall and wet–dry cycling conditions, water penetrating into the pore network can facilitate the migration of chloride ions and heavy metals, consequently leading to contaminant release, pore structure deterioration, matrix cracking, and reinforcement corrosion [25,26,27,28,29,30]. Moreover, the rough surface morphology and high water absorption capacity of fly ash particles can increase the moisture sensitivity of mortar systems and reduce their impermeability and durability when directly incorporated. Therefore, hydrophobic modification of fly ash is necessary to introduce low-surface-energy functional groups or form hydrophobic layers, thereby reducing particle hydrophilicity, suppressing capillary water absorption, and blocking the continuous transport of water and aggressive ions, ultimately mitigating heavy metal release and material degradation [31]. However, a direct comparison between carbonation modification and hydrophobic modification within the same sulfoaluminate cement system remains insufficiently explored. Therefore, this study prepared sulfoaluminate cement mortars incorporating hydrophobically modified and carbonated MSWI fly ash and systematically compared the effects of the two modification strategies on mechanical properties, hydration heat evolution, water absorption, microstructural characteristics, and molecular-scale interaction mechanisms.

## 2. Materials and Methods

### 2.1. Materials

**Figure 1:**
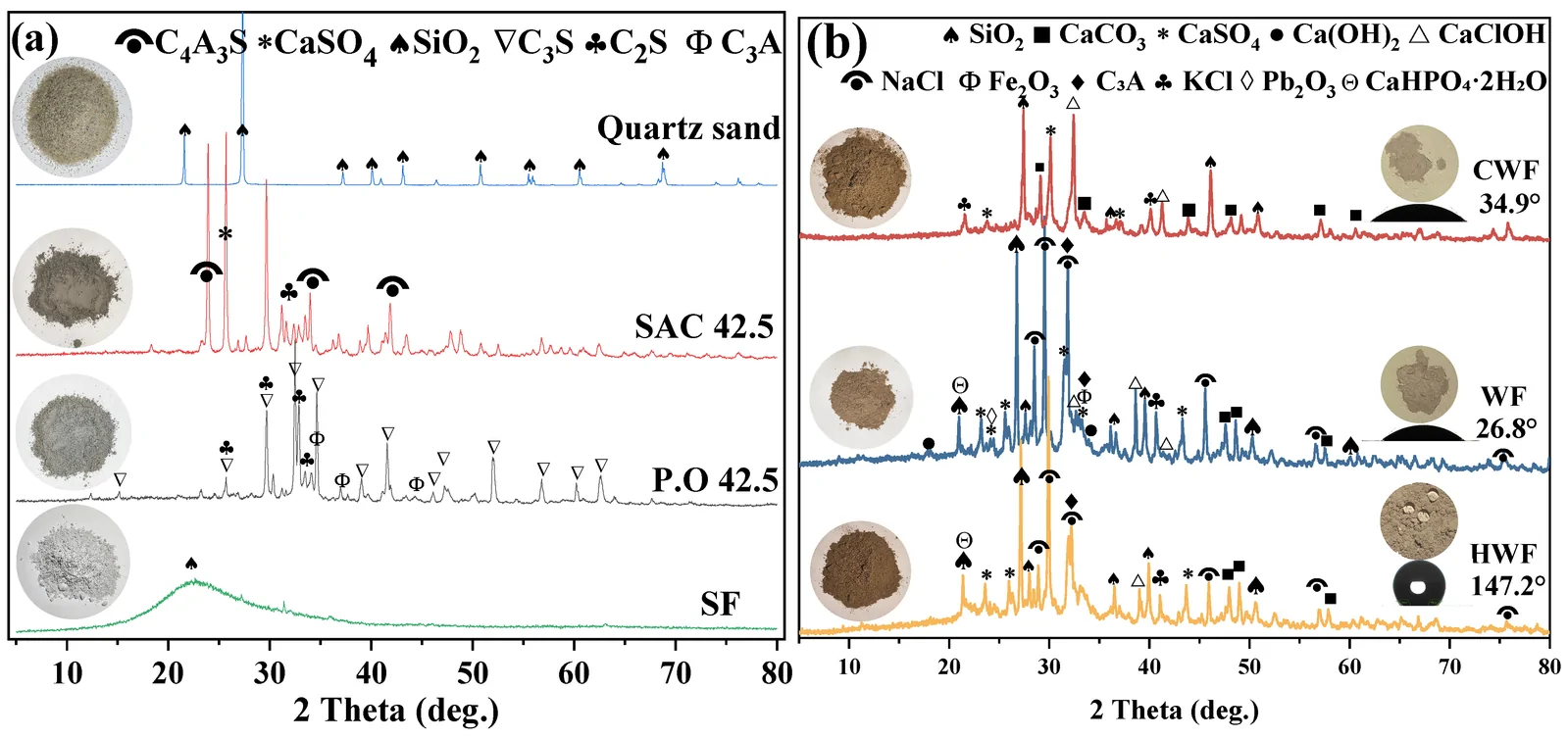
Morphologies and XRD characterization of the raw materials: (**a**) morphologies and XRD patterns of quartz sand, SAC 42.5, P.O 42.5 cement, and silica fume; (**b**) XRD patterns, static water contact angles, and corresponding water-droplet images of CWF, WF, and HWF.

R·SAC 42.5, P.O 42.5 cement, silica fume (SF), and different types of MSWI fly ash were used as cementitious materials, with quartz sand used as the fine aggregate, to construct an SAC–Portland cement–silica fume composite binder system. The MSWI fly ash included untreated fly ash (WF), hydrophobically modified fly ash (HWF), and water-washed–carbonated fly ash (CWF). In this system, SAC promotes the rapid formation of aluminate hydration products such as ettringite (AFt), Portland cement supplies Ca and Si species for the formation of C-S-H, while silica fume participates in subsequent pozzolanic reactions through its reactive SiO_2_ and contributes to microstructural refinement. This composite binder therefore provides both sulfoaluminate- and silicate-based hydration environments, enabling comparison of the effects of WF, HWF, and CWF on hydration, microstructure, and fly ash–C-S-H interfacial behavior. R·SAC 42.5, P·O 42.5 cement, and quartz sand were supplied by Zhengzhou Jingwei Building Materials Co., Ltd., Zhengzhou, China. SF was provided by Luoyang Yumin Microsilica Powder Co., Ltd., Luoyang, China. The original fly ash (WF) was collected from Xingjin Waste Incineration Plant in Zhengzhou, China, and subsequently modified to prepare HWF and CWF. The main mineral phases of SAC consisted of ye’elimite (C_4_A_3_S, 56.2%) and belite (C_2_S, 26.2%). The major phases of ordinary Portland cement included alite (C_3_S, 57.3%), belite (C_2_S, 23.5%), tricalcium aluminate (C_3_A, 6.5%), and tetracalcium aluminoferrite (C_4_AF, 10.5%). The morphologies and X-ray diffraction (XRD) patterns of the raw materials, together with the static water contact angles and corresponding water-droplet images of the three types of MSWI fly ash, are presented in Figure 1, while their chemical compositions are summarized in Table 1. The main mineral phases in the original fly ash included SiO_2_, calcium-containing phases, and chloride-containing compounds. The static water contact angle of WF was 26.8°, indicating a distinctly hydrophilic surface. After water-washing–carbonation treatment, the chloride-related diffraction peaks became less pronounced because part of the soluble salts were removed during the water-washing process, whereas the characteristic peaks of carbonates, gypsum, and SiO_2_ became more prominent. The static water contact angle of CWF was 34.9°, indicating that it remained hydrophilic. In contrast, the static water contact angle of HWF reached 147.2°, which was substantially higher than those of WF and CWF, demonstrating that MTMS modification markedly reduced the surface wettability of the fly ash and imparted pronounced hydrophobic characteristics.

### 2.2. Methods and Procedures

**Figure 2:**
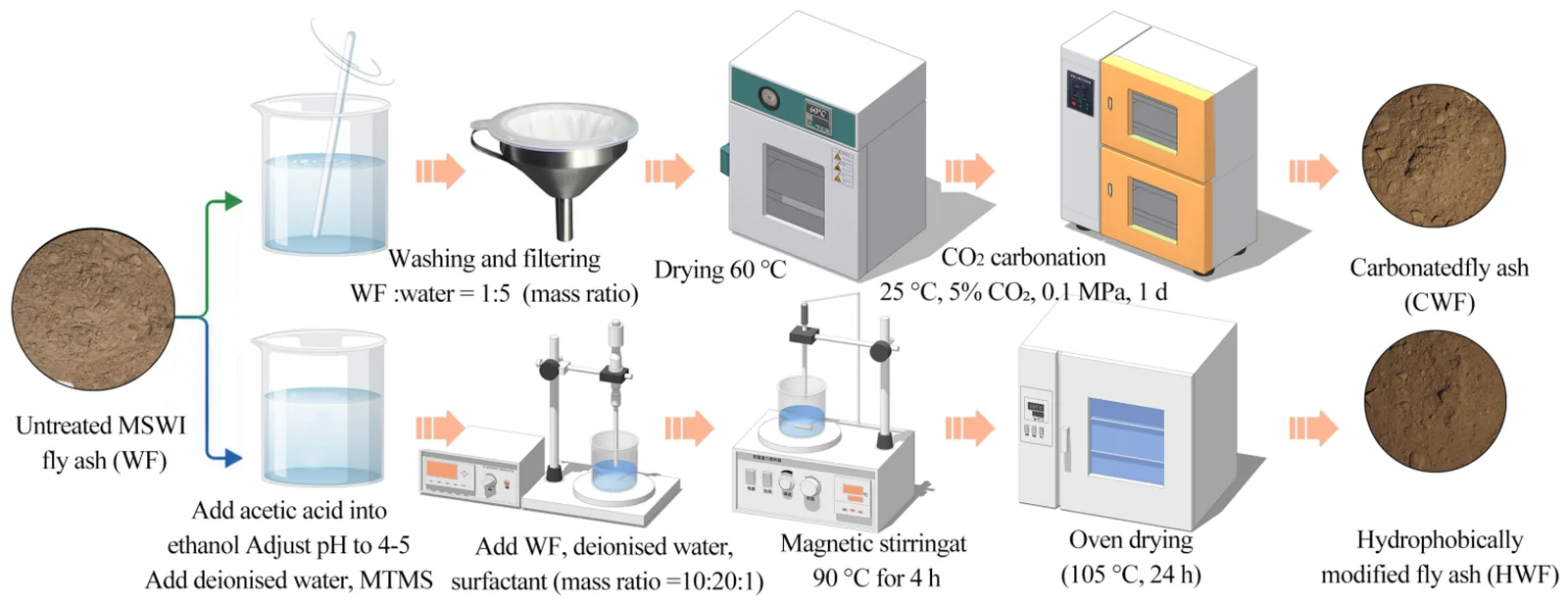
Flowchart of hydrophobic and carbonation modification processes for waste fly ash.

The mix proportions of the mortars incorporating modified fly ash are presented in Table 2. The mass ratio of cementitious materials to fine aggregate was fixed at 1:1, with a water-to-binder ratio of 0.3. The fly ash content was set at 15% of the total cementitious materials. The modification procedures for fly ash are illustrated in Figure 2. For the water-washing–carbonation treatment, the MSWI fly ash was first mixed with water at a mass ratio of 1:5 and subjected to water washing. The washed fly ash was then dried in an oven at 60 °C and subsequently placed in a carbonation chamber. The carbonation process was conducted at 25 °C with a CO_2_ concentration of 5%, a pressure of 0.1 MPa, and a duration of 1 day. It should be noted that the preparation of carbonated MSWI fly ash involved a water-washing pretreatment step. Water washing removed part of the soluble salts and chlorides, which was associated with weakened Friedel’s salt-related characteristic peaks and reduced local Cl content. The subsequent carbonation process promoted CaCO_3_ formation and influenced hydration reactions and pore structure development through heterogeneous nucleation, micro-filling, and mineral phase regulation. Because no separate water-washing-only control group was included in this study, the individual contributions of water washing and carbonation to hydration, mechanical properties, and Cl-related behavior could not be quantitatively distinguished. Therefore, the results for CWF are interpreted as the combined effects of the water-washing–carbonation treatment, and the discussion and conclusions avoid attributing these effects solely to carbonation.

For hydrophobic modification, acetic acid was first added dropwise into an ethanol solution to adjust the pH value to 4–5. Deionized water and methyltrimethoxysilane (MTMS) were then added and mixed thoroughly to promote silane hydrolysis. Subsequently, MSWI fly ash, deionized water, and surfactant were added at a mass ratio of 10:20:1. The mass ratio of MTMS to fly ash was 1:5, and Triton X-100 with a purity of 98% was used as the surfactant. The mixture was continuously stirred at 90 °C for 4 h using a magnetic stirrer to promote the interaction between the hydrolyzed silane species and the fly ash surface. The treated fly ash was then dried at 105 °C to obtain hydrophobically modified fly ash (HWF).

The aim of this study was to compare the effects of untreated, water-washed–carbonated, and hydrophobically modified MSWI fly ash under the same binder system and total MSWI fly ash content. Therefore, the contents of SAC, P.O 42.5 cement, silica fume, the water-to-binder ratio, the sand-to-binder ratio, and the total MSWI fly ash dosage were kept constant, while WF was progressively replaced by CWF or HWF to minimize interference from changes in the binder system and highlight the effects of fly ash modification and replacement level.

### 2.3. Material Performance Tests

The compressive strength of the mortars was measured according to the Chinese standard GB/T 17671-2021 [32]. The compressive strength of each mixture was tested using three replicate specimens, and the results are expressed as the mean ± standard deviation. One-way analysis of variance (ANOVA) was used to evaluate differences in compressive strength among mortar mixtures at the same curing age, followed by Tukey’s multiple comparison test for pairwise comparisons. A value of *p* < 0.05 was considered statistically significant. The water absorption test was conducted following GB/T 50081-2019 [33]. The hydration heat was measured in accordance with ASTM C1679 [34]. Cement pastes with different mix proportions were prepared according to Table 2. The heat evolution of the cement pastes was determined using an isothermal conduction calorimeter (TAM Air, TA Instruments, New Castle, DE, USA). The measurements were conducted at a constant temperature of 20 °C for 72 h. The measurements were performed using an internal admix ampoule. The cementitious materials and mixing water were placed separately in the calorimetric channel before mixing and allowed to reach thermal equilibrium at the testing temperature. Hydration was then initiated using the internal mixing device, and the onset of internal mixing was defined as time zero. Because mixing was carried out inside the calorimeter after thermal equilibration, early heat loss and baseline disturbances associated with external mixing, sample transfer, and thermal re-equilibration were minimized, allowing the rapid heat evolution during the initial hydration stage to be recorded. Each sample was tested in an independent calorimetric channel.

For the water absorption measurement, mortar specimens cured for 28 days were first dried to a constant mass. Subsequently, the specimens were completely immersed in water, with the water level maintained at 25 mm above the top surface of the samples. The mass changes of the specimens were continuously recorded at different immersion times over a period of 5 days. To visually evaluate the wettability characteristics of different modified MSWI fly ashes and the corresponding mortar surfaces, water-droplet wetting morphology was observed. After curing to the specified age, the mortar specimens were uniformly polished, and equal volumes of deionized water were dropped onto the test surfaces under identical conditions to record the macroscopic droplet morphology. The environmental conditions, droplet volume, and image-capture time were kept consistent for all specimens to enable a qualitative comparison of the surface wetting behavior among different mixtures.

The microstructural morphology of the mortars was examined using scanning electron microscopy (SEM, TESCAN MIRA LMS, Brno, Czech Republic), and the elemental composition on the mortar surfaces was analyzed by energy-dispersive spectroscopy (EDS). Prior to SEM observation, the specimen surfaces were sputter-coated with Pt to improve electrical conductivity. During EDS quantification, Pt was treated as an externally introduced coating element and excluded from the quantitative analysis, while the weight percentages (wt%) and atomic percentages (atomic%) of the remaining detected elements were normalized to 100%. EDS was primarily used to characterize the local elemental composition of selected regions and provides semi-quantitative results. Therefore, the EDS data were used only to compare local elemental distribution characteristics among different specimens and were not regarded as a quantitative measure of the overall chloride content or chloride-ion migration capacity of the specimens. For X-ray diffraction (XRD) analysis, mortar samples were ground into powders with particle sizes below 75 μm. The XRD patterns were collected over a 2θ range of 10–80° at a scanning rate of 0.5°/min.

The pore structure of the mortar was characterized using a low-field nuclear magnetic resonance instrument (NIUMAG MesoMR12-060, Suzhou Niumag Analytical Instrument Corporation, Suzhou, China). The NMR signal intensity is related to the water content of the sample. The transverse relaxation time (T_2_) of the mortar was measured using a Carr–Purcell–Meiboom–Gill (CPMG) pulse sequence, as expressed in Equation (1) [35].

(1) $$\frac{1}{T_{2}}\approx \frac{1}{T_{2s}}=\rho \frac{S}{V}=\frac{\rho F_{s}}{R}=\frac{2\rho }{R}$$

In the equation, T_2_s is the surface relaxation time; ρ is the surface relaxivity, taken as 12 nm/ms [36]; S/V is the ratio of pore surface area (S) to fluid volume (V); R is the pore radius; and F_s_ is the pore geometry factor. Assuming cylindrical pores, F_s_ was taken as 2.

To elucidate the interfacial interaction mechanisms between modified fly ash and hydration products, molecular dynamics (MD) simulations were performed by constructing fly ash–C-S-H interface models. First, representative molecular models of WF, HWF, and CWF were established based on their major chemical compositions obtained from experimental characterization.

The WF, HWF, and CWF models developed in this study were constructed as representative atomistic interface models constrained by the XRF elemental composition, XRD phase identification, and the corresponding modification processes, with the aim of comparing the relative effects of different modification methods on fly ash–C-S-H interfacial interactions. Accordingly, the emphasis was placed on the relative differences among the three systems under identical modeling and computational conditions, rather than regarding the constructed models as unique representations of the actual fly ash surfaces.

The WF model was constructed based on the major mineral phases identified by XRD and the elemental composition determined by XRF. Si, Al, and Ca were used to represent the aluminosilicate–calcium inorganic framework of the fly ash, while Na and Cl were introduced as corresponding ionic species to represent the soluble salt components present in untreated fly ash. The model was first constructed as a periodic structure and geometrically optimized, after which a surface along a selected crystallographic direction was generated for contact with C-S-H. The HWF model was developed based on the inorganic structure of WF, with methylsiloxane structures derived from MTMS hydrolysis introduced as the hydrophobic modification component. These structures were connected to surface silanol sites through Si–O–Si bonds to represent the condensation reaction between hydrolyzed MTMS species and hydroxyl groups on the fly ash surface. The CWF model was also based on the WF inorganic framework. According to the conversion of CaO/Ca(OH)_2_ to CaCO_3_ during carbonation, CaCO_3_-related structures were introduced at the surface, while the proportions of soluble salt species such as Na and Cl were reduced according to the experimentally determined composition of CWF to represent the surface chemical environment after the water-washing–carbonation treatment.

It should be noted that MSWI fly ash is a multiphase material with pronounced chemical and structural heterogeneity, and its actual surface cannot be fully represented by a single atomistic model. Therefore, the WF, HWF, and CWF models established in this study should be regarded as representative interface models constructed on the basis of experimentally determined compositions and major phases. Their primary purpose is to compare the relative effects of different modification methods on C-S-H interfacial interactions and component migration behavior under consistent simulation conditions, rather than to reproduce all possible atomic configurations of actual fly ash surfaces. The molecular dynamics results obtained from these models were therefore mainly used to interpret the relative trends observed experimentally.

The C-S-H model used in this study was constructed following the methods proposed by Pellenq and Hou [37,38]. An 11 Å tobermorite structure was adopted as the initial model, in which the silicate chains were cleaved and partially removed at the bridging sites to generate a chain-length distribution more representative of realistic C-S-H gel. The original ordered water molecules were removed, and the monoclinic unit cell was converted into an orthorhombic cell. Subsequently, saturated water molecules were introduced into the interlayer region using the grand canonical Monte Carlo (GCMC) method, followed by relaxation under the NPT ensemble until equilibrium was reached. The resulting C-S-H model had a density of 2.45 g/cm^3^ and a Ca/Si ratio of 1.67, which are in good agreement with previously reported experimental and simulation results [39,40]. It should be noted that, apart from the interlayer water introduced into C-S-H by GCMC, no additional explicit water molecules were added to the external fly ash–C-S-H contact region. Therefore, the present model was primarily intended to characterize the intrinsic solid–solid interfacial interactions between different modified fly ashes and C-S-H, as well as the relative local mobility of interfacial species, rather than to directly simulate ionic diffusion under saturated pore-solution conditions.

All molecular dynamics simulations were performed using the Forcite module in Materials Studio 2024. The COMPASS II force field was consistently employed to describe the interactions among inorganic phases, ions, water molecules, carbonates, and organic methylsiloxane groups. Atomic partial charges were assigned according to the atom types and charge parameters implemented in the COMPASS II force field. Three-dimensional periodic boundary conditions were applied. After geometry optimization, the systems were equilibrated in the NVT ensemble at 298 K using a Nosé thermostat. A time step of 1 fs was employed. The equilibration and production stages were conducted for 2000 ps and 1000 ps, respectively, and trajectory frames were saved every 1 ps for subsequent analyses of interfacial interaction energy and mean square displacement (MSD). van der Waals interactions were calculated using the atom-based method, while long-range electrostatic interactions were evaluated using the particle–particle particle–mesh (PPPM) method with an accuracy of 0.001 kcal/mol.

**Figure 3:**
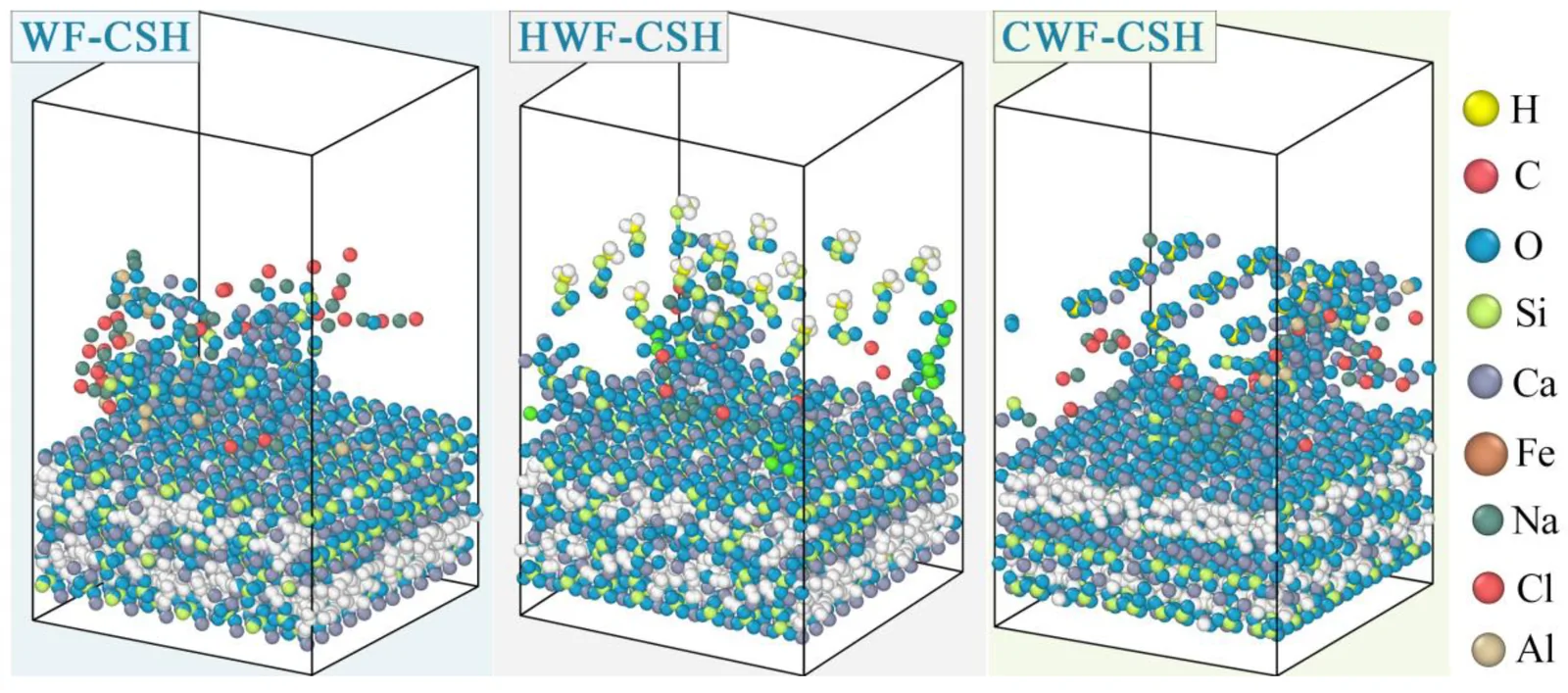
Molecular dynamics simulation models of three types of fly ash–C-S-H interfaces.

Subsequently, the three fly ash models were individually assembled with the C-S-H gel model to construct three composite interface systems, namely WF–C-S-H, HWF–C-S-H, and CWF–C-S-H, through interfacial contact, as illustrated in Figure 3. After model construction, geometry optimization was initially performed to eliminate unfavorable atomic contacts and achieve a reasonable initial configuration. Subsequently, molecular dynamics equilibration was conducted to stabilize the constructed interface systems.

Based on the equilibrated trajectory files, the interfacial interaction energy and its energy components, including van der Waals and electrostatic interactions, were calculated to evaluate the interfacial bonding characteristics between different fly ash types and C-S-H. Meanwhile, the mean square displacement (MSD) and diffusion coefficient were employed to characterize the migration behavior of various components within the interface systems, thereby elucidating the effects of carbonation and hydrophobic modification on the fly ash–C-S-H interfacial interactions and stability at the molecular level.

At the molecular scale, the key interactions governing repulsive and attractive forces include covalent bonds, electrostatic interactions, and van der Waals forces [41]. The interfacial energy represents the interaction energy between molecules. A positive interfacial energy indicates repulsive interactions between two molecular components, whereas a negative value indicates attractive interactions. The interfacial interaction energy can be calculated using Equation (2) [42].

(2) $$E_{\mathrm{int}}=E_{\mathrm{total}}-E_{C\text{-}S\text{-}H}-E_{F}$$

where E_int_ represents the interfacial interaction energy between C-S-H and fly ash; E_C−S−H_ is the total potential energy of the isolated C-S-H model; E_F_ denotes the total potential energy of the isolated fly ash models, including WF, HWF, and CWF; and E_total_ is the total potential energy of the combined C-S-H/fly ash interface system. The binding energy per unit area (E_b,area_) was calculated by normalizing the total binding energy with respect to the projected interfacial contact area:

(3) $$E_{b,\mathrm{area}}=\frac{E_{b}}{A}$$

In Equation (3), E_b,area_ represents the binding energy per unit area, E_b_ denotes the total binding energy (E_b_ = E_int_), and A represents the projected interfacial contact area. The dimensions of the interfaces in the three models were identical, with a size of 44.6 Å × 43.8 Å, corresponding to a projected interfacial area of 1953.5 Å^2^. The calculated binding energies were uniformly converted into J/m^2^.

The mean square displacement (MSD) was used to describe the deviation between the particle position at a given time and its initial position [43]. After completing the 2000 ps NVT equilibration, each system was subjected to a further 1000 ps production simulation. The MSD–time relationship over the 0–1000 ps production trajectory was linearly fitted using the least-squares method, and the goodness of fit was evaluated using the coefficient of determination (R^2^). The diffusion coefficient was calculated from the slope of the fitted interval, and its uncertainty was obtained by propagating the standard error of the regression slope and expressed as a 95% confidence interval. The calculation formula is given in Equation (4).

(4) $$MSD(t)=\langle |r_{i}(t)-r_{i}\left(0\right){|}^{2}\rangle$$

where r_i_(t) represents the displacement vector of molecule i at time t, and r_i_(0) represents the initial displacement vector of molecule i at the beginning of the simulation.

Furthermore, the diffusion coefficient can be determined from the linear relationship between MSD and simulation time [44]. The diffusion coefficient was calculated according to Equation (5).

(5) $$\mathrm{Diffusion}\;\mathrm{coefficient}=\underset{t\to \infty }{\lim}\frac{\left\langle |r_{1}(t)-r_{1}\left(0\right){|}^{2}\right\rangle }{6t}=\underset{t\to \infty }{\lim}\frac{\mathrm{MSD}(t)}{6t}=\frac{m}{6}$$

where t represents the simulation time, MSD(t) denotes the mean square displacement at time t, and m represents the slope of the MSD–time curve.

A multiscale approach was used to investigate Cl-related species and their interfacial migration behavior in MSWI fly ash. XRF and XRD characterized the overall chemical composition and chloride-bearing phases, SEM–EDS examined local Cl distribution, and water absorption and NMR evaluated water ingress and water-accessible pore structure. MSD analysis in MD simulations was used to compare the relative local mobility of Cl and Na under identical conditions. These methods were used to relate composition, phase assemblage, pore structure, and interfacial species mobility, rather than to replace macroscopic chloride migration tests.

## 3. Results and Discussion

### 3.1. Compressive Strength

**Figure 4:**
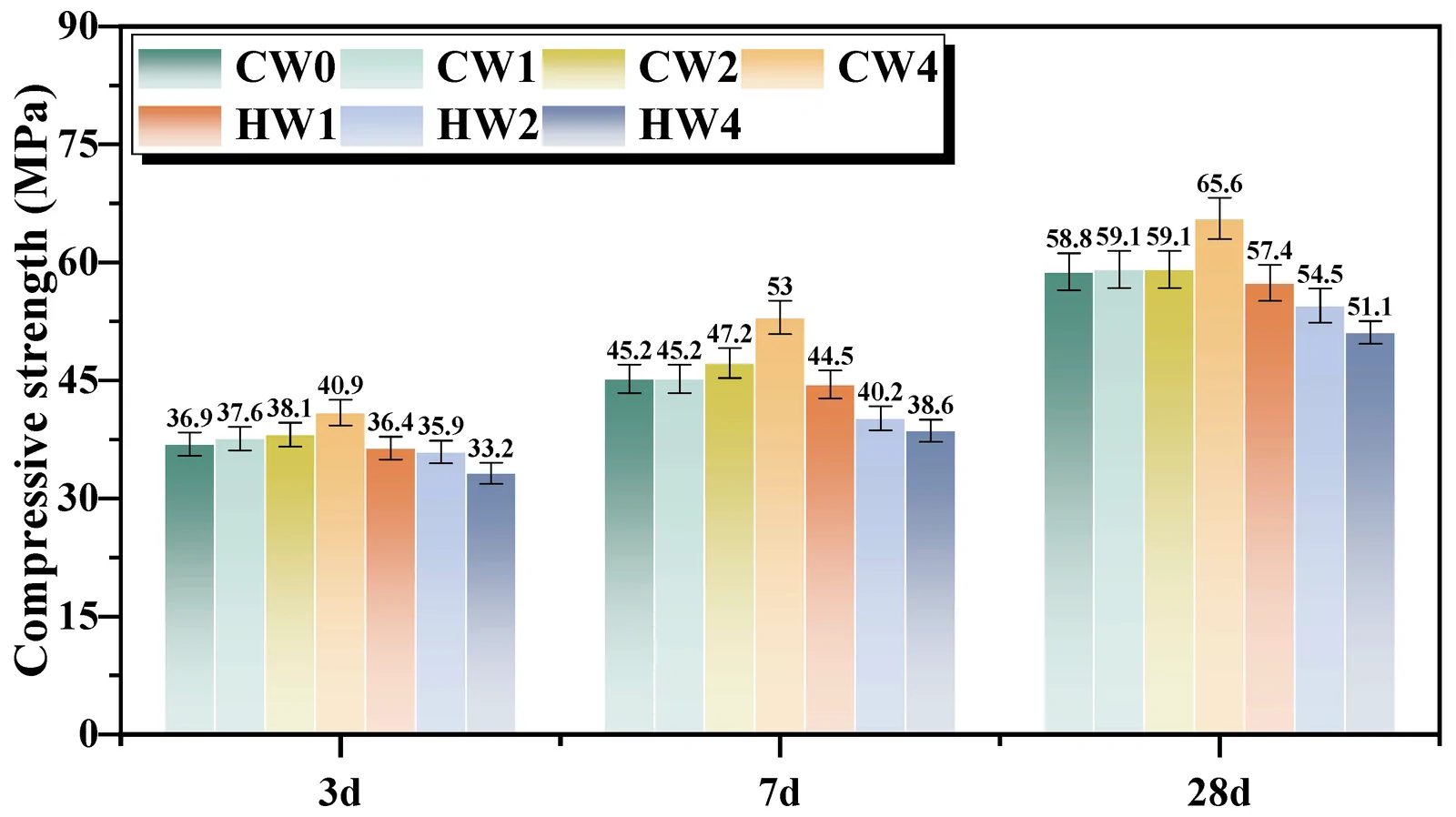
Compressive strength of mortars with different mix proportions.

The compressive strengths of mortars with different mix proportions are presented in Figure 4. With increasing CWF replacement, the compressive strength of the CW-series mortars showed an increasing trend. At 28 days, the maximum increase in compressive strength was 6.8 MPa compared with the reference group. This improvement may result from the combined effects of desalination during water washing and subsequent carbonation mineralization. Water washing can remove part of the soluble salts and thereby reduce their potential interference with binder hydration, while the CaCO_3_ formed during subsequent carbonation can provide heterogeneous nucleation sites and a micro-filling effect, favoring hydration-product formation and local microstructural refinement. Combined with the XRD, SEM, and NMR results, the CaCO_3_ formed after water-washing–carbonation treatment may contribute to microstructural refinement and strength development through these effects.

In contrast, the compressive strength of the HW-series mortars generally decreased with increasing HWF replacement, with a maximum reduction of 7.7 MPa at 28 days relative to the reference group. This decrease is mainly associated with the reduced surface wettability of the particles after hydrophobic modification. The low-surface-energy organic layer formed by MTMS modification can weaken the contact of water with fly ash particles and reactive sites, thereby limiting the dissolution of some components and the continuous growth of hydration products [23]. In addition, interlayer and confined water in C-S-H gel participates in hydrogen-bond networks and interparticle cohesive interactions, and changes in its state may further affect the structural stability of the gel and the macroscopic mechanical properties [45].

### 3.2. Hydration Heat

**Figure 5:**
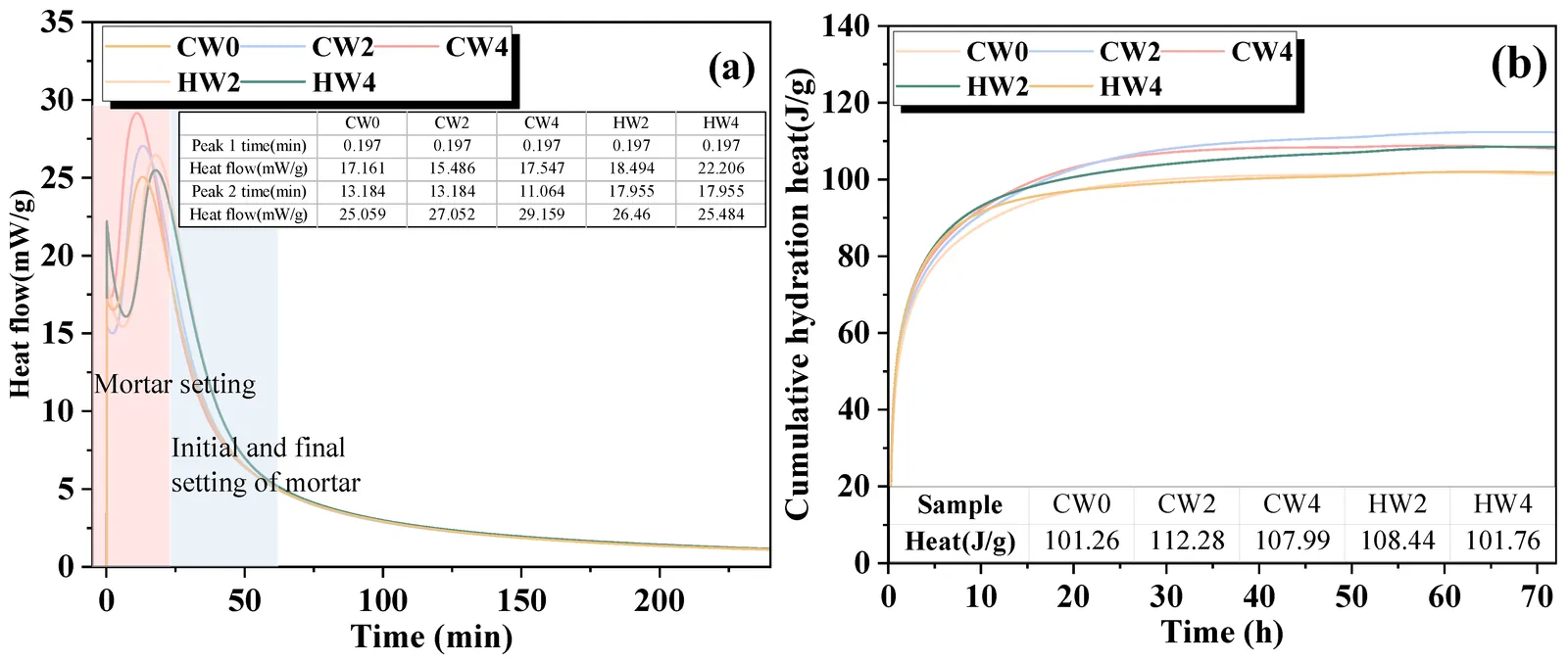
Hydration heat of mortars with different mix proportions: (**a**) heat flow; (**b**) cumulative heat release.

The hydration heat results of pastes with different mix proportions are presented in Figure 5. Different modified fly ash proportions significantly affected the early-age hydration behavior of the composite cementitious systems. All samples exhibited two distinct exothermic peaks. The first peak was mainly associated with the initial wetting of cement particles, rapid dissolution of ye’elimite, and the release of soluble components from fly ash, whereas the second peak corresponded to the accelerated formation of hydration products, including AFt, AH_3_, and C-S-H [46].

Compared with CW0, the second exothermic peak of CW4 occurred earlier, shifting from 13.18 min to 11.06 min, while the peak intensity increased from 25.06 mW/g to 29.16 mW/g. Meanwhile, the cumulative heat release increased from 101.26 J/g to 107.99 J/g. This indicates that CWF after the water-washing–carbonation treatment is beneficial to early-age hydration. This effect may arise from the combined removal of soluble salts during water washing and the subsequent formation of CaCO_3_ during carbonation, with CaCO_3_ providing heterogeneous nucleation sites and promoting the formation of early hydration products [11]. In contrast, the second exothermic peak of HW4 was delayed to 17.96 min, and the cumulative heat release decreased to 101.76 J/g, indicating that hydrophobic fly ash restricted water transport and ion diffusion, thereby inhibiting the main hydration reactions and sustained heat release at later stages. The improvements in hydration and strength observed for water-washed–carbonated fly ash in this study are generally consistent with previous studies [12,13,14]. The present study further elucidates the underlying mechanism in the composite binder system through combined analyses of hydration heat and microstructure.

### 3.3. Water Absorption

**Figure 6:**
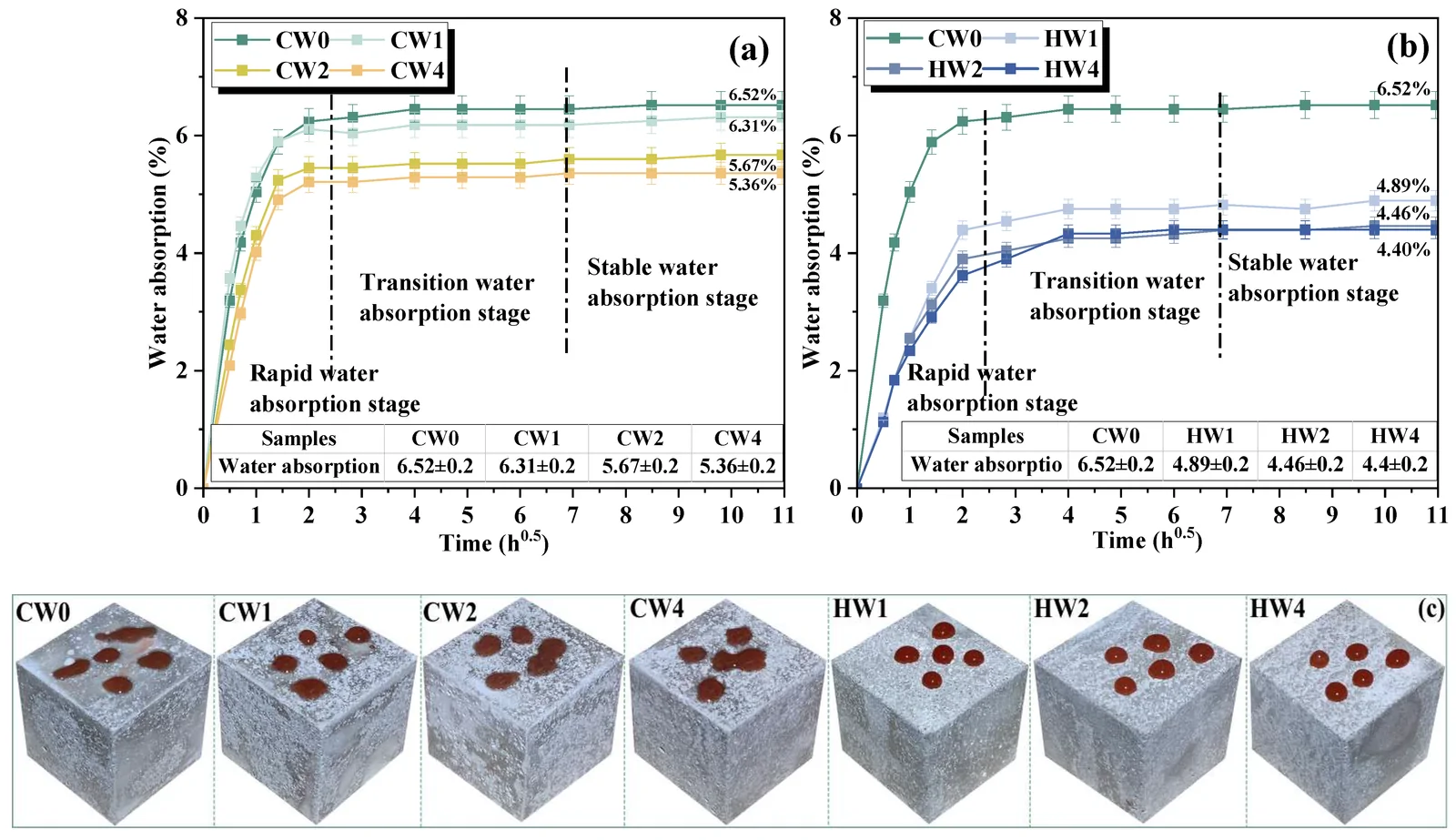
Water absorption and surface wettability of mortars with different mix proportions: (**a**) and (**b**) water absorption; (**c**) macroscopic water-droplet morphology.

The water absorption and surface wetting morphology of mortars with different mix proportions are presented in Figure 6. MSWI fly ash exhibits a loose, porous, and heterogeneous structure with a high specific surface area, resulting in strong water absorption capacity. This characteristic increases the water demand of the cementitious system and reduces its flowability [25]. With increasing replacement ratios of CWF, the water absorption of the mortars gradually decreased. When the replacement ratios of CWF were 16.7%, 33.3%, and 66.7%, the water absorption decreased by 3.2%, 13.0%, and 17.8%, respectively. The incorporation of CWF promoted hydration and mineralization reactions, generating cementitious products that filled internal pores and reduced pore connectivity, thereby suppressing water transport and absorption. The NMR results showed that the porosity decreased from 6.27% for CW0 to 5.99% for CW2 and 4.84% for CW4, providing quantitative evidence of pore-structure refinement in the water-washed–carbonated groups.

With increasing replacement ratios of HWF, the water absorption of mortars decreased significantly and then tended to stabilize. When the replacement ratios of HWF were 16.7% (HW1), 33.3% (HW2), and 66.7% (HW4), the water absorption decreased by 25.0%, 31.6%, and 32.5%, respectively. The reduction in mortar water absorption by HWF is consistent with previous studies on silane-based hydrophobic treatment [15]. Unlike the direct incorporation or surface application of hydrophobic agents, this study pre-modified MSWI fly ash particles with MTMS to regulate the wettability and water transport behavior of the mortar. Compared with CWF, HWF exhibited a more pronounced reduction effect on water absorption. This improvement can be attributed to the interaction between silicon-containing components in fly ash and methylsiloxane hydrophobic groups, which promotes the formation of hydrophobic films within the pore structure, thereby reducing the surface wettability of the mortar and its water adsorption [47].

To visually evaluate the wetting characteristics of different modified MSWI fly ashes and the corresponding mortar surfaces, water-droplet wetting morphology was observed. As the HWF replacement level increased, the extent of water-droplet spreading on the mortar surface decreased noticeably, indicating that MTMS-modified fly ash reduced the surface wettability of the mortar. This observation is consistent with the lower water absorption of the HW-series mortars, suggesting that the improvement in water resistance was associated not only with pore-structure changes but also with enhanced hydrophobicity.

### 3.4. NMR

**Figure 7:**
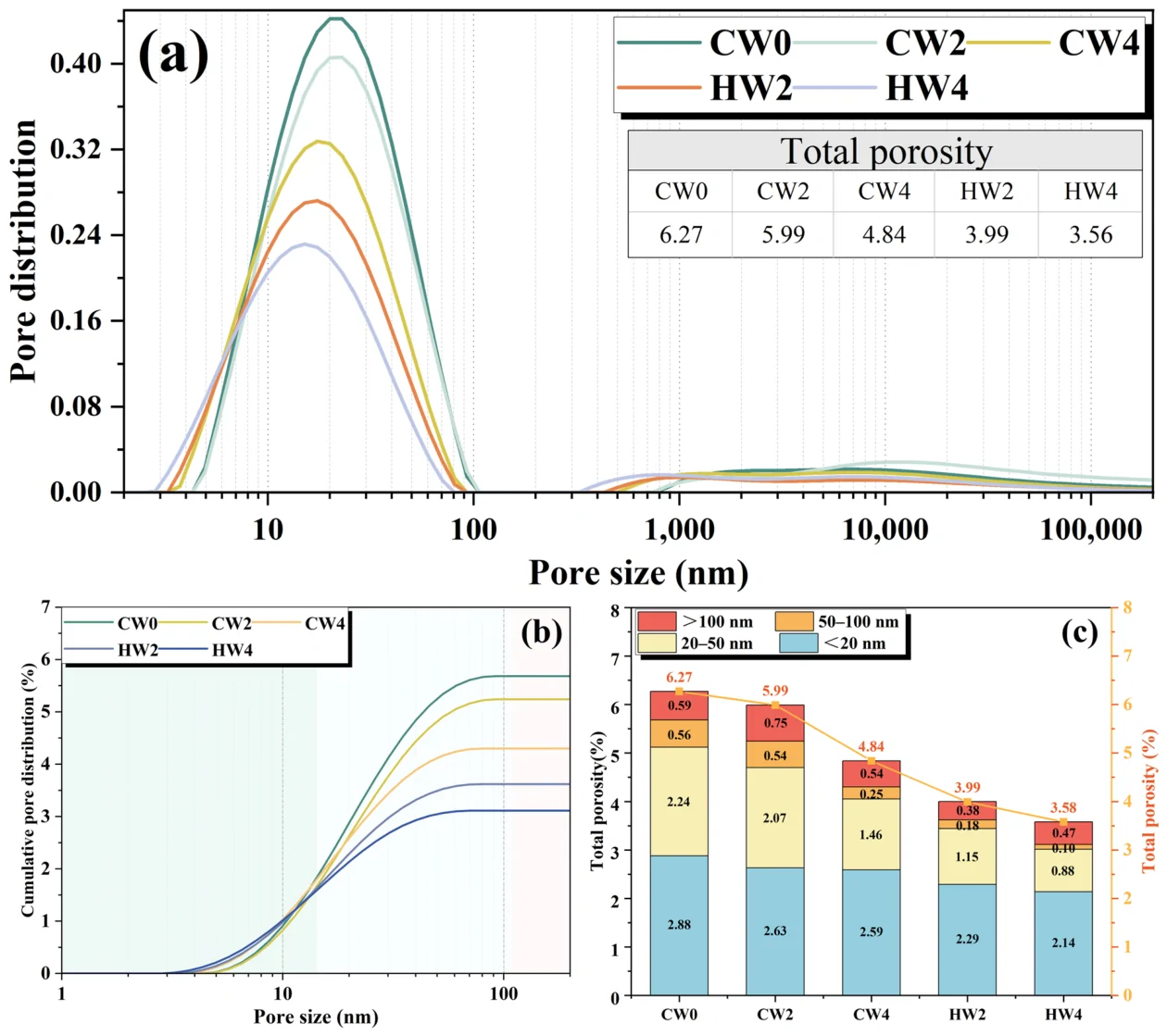
Pore structure characteristics of mortars with different mix proportions: (**a**) pore-size distribution and total porosity; (**b**) cumulative pore-size distribution; (**c**) porosity distribution in different pore-size ranges.

The NMR results of mortars with different mix proportions are shown in Figure 7. CW0 exhibited the highest porosity of 6.27%, indicating that untreated MSWI fly ash led to a more porous mortar structure. This can be attributed to the loose and porous morphology, rough surface, and high water absorption of the raw fly ash particles, which can increase the water demand of the paste and result in a less compact structure [48]. With increasing CWF replacement, the total porosity decreased from 6.27% for CW0 to 5.99% for CW2 and 4.84% for CW4, indicating that the combined water-washing–carbonation treatment effectively improved the pore structure of the mortar. This change may result from the combined effects of desalination during water washing and subsequent carbonation mineralization. In particular, the CaCO_3_ formed during carbonation can provide heterogeneous nucleation sites and exert a micro-filling effect, thereby promoting hydration-product accumulation and local pore-structure refinement [49]. This result is consistent with the enhanced CaCO_3_-related diffraction features observed by XRD.

From the pore-size distribution, CW0 exhibited the highest peak intensity in the main pore-size region and a relatively pronounced tail in the larger-pore region, indicating not only a higher total porosity but also a broader and more heterogeneous pore structure. After water-washing–carbonation treatment, the main peak of CW4 decreased markedly, suggesting an effective reduction in the dominant pore population. As shown in Figure 7c, the proportion of 20–50 nm pores decreased from 2.24% in CW0 to 1.46% in CW4, while the proportions of 50–100 nm and >100 nm pores decreased from 0.56% to 0.25% and from 0.59% to 0.54%, respectively. These quantitative results indicate that the water-washing–carbonation treatment particularly reduced the fraction of pores in the 20–100 nm range, further supporting the possible micro-filling effect of CaCO_3_ and the associated refinement of the pore structure.

HW2 and HW4 exhibited total porosities of 3.99% and 3.58%, respectively, both lower than that of CW0. In terms of pore structure, the HW series showed decreases across all pore-size ranges. In particular, the proportion of 20–50 nm pores decreased from 2.24% in CW0 to 1.15% and 0.88% in HW2 and HW4, respectively, while the proportion of pores larger than 100 nm decreased to approximately 0.38–0.47%. Because NMR primarily relies on the hydrogen-nuclei signal from pore water, hydrophobic modification can reduce the surface free energy of hydration products and pore walls, weaken pore-wall wettability, and restrict water penetration into part of the pore system. This may suppress continuous water transport and the migration of aggressive species through the pore network, while also limiting pore expansion associated with subsequent moisture migration, thereby inhibiting the development of a connected capillary pore network [50]. These results are consistent with the lower water absorption observed in the HW groups.

### 3.5. XRD

**Figure 8:**
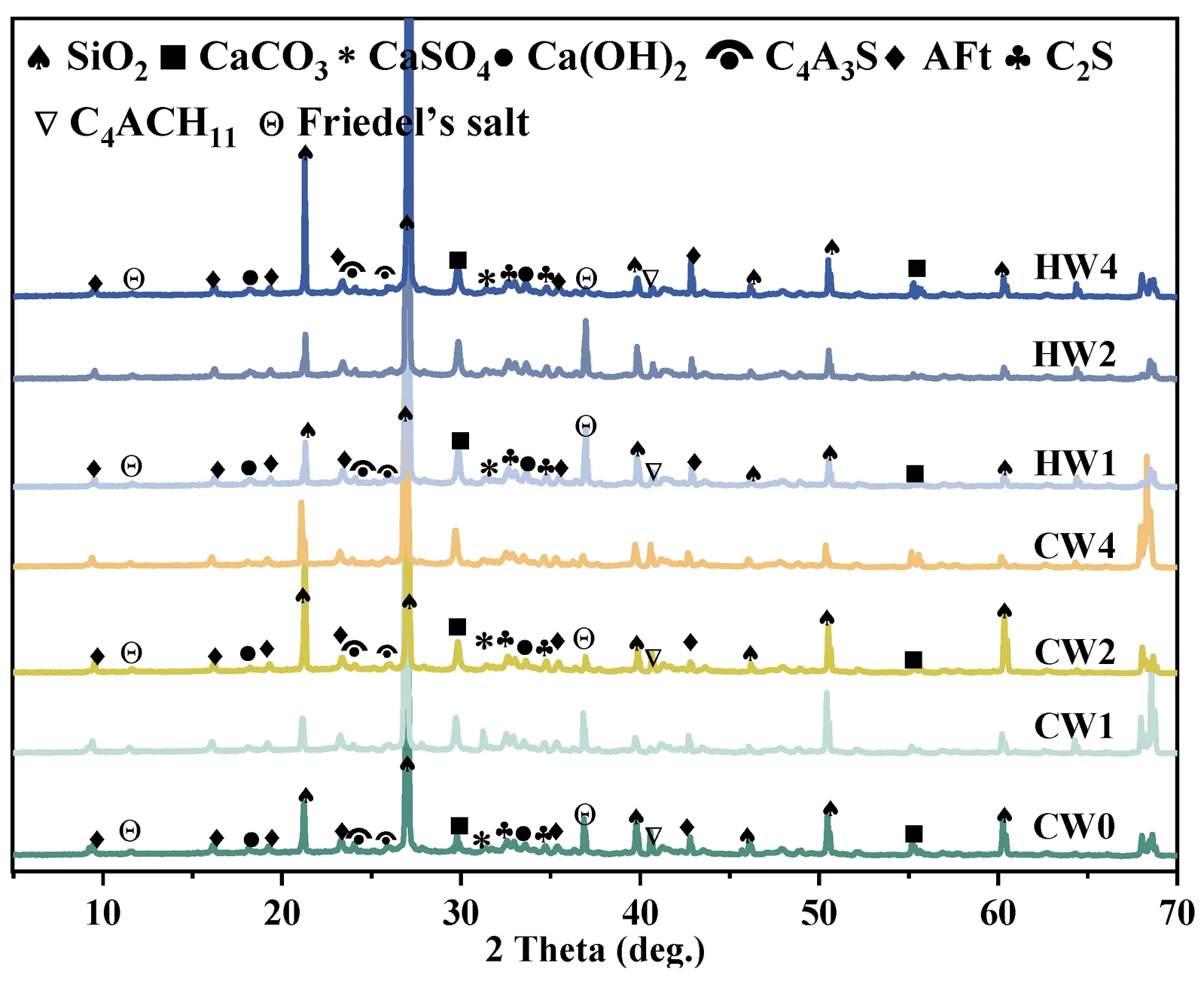
XRD patterns of mortars with different mix proportions.

The XRD patterns of mortars incorporating different fly ash contents are presented in Figure 8. The main crystalline phases identified in all samples included SiO_2_, CaSO_4_, CaCO_3_, Ca(OH)_2_, AFt, Friedel’s salt, unhydrated C_2_S, and a small amount of carbon-containing AFm phases. With increasing replacement content of carbonated MSWI fly ash, the characteristic diffraction peaks of CaCO_3_ in the CW series gradually became more pronounced, while the diffraction peaks corresponding to Ca(OH)_2_ and AFt remained observable. Compared with CW0, the diffraction features of CaCO_3_ and monocarbonate phases became more evident in CW1, CW2, and CW4, which was mainly attributed to the introduction of pre-existing CaCO_3_ from CWF. In addition to acting as heterogeneous nucleation sites and micro-filling components, CaCO_3_ can react with C_4_A_3_S in cement and Ca(OH)_2_ generated during hydration, promoting the formation of monocarbonate phases (C_3_A·CaCO_3_·11H_2_O, C_4_ACH_11_) [51].

The presence of AFt and Friedel’s salt was detected in all mortar samples, indicating that the sulfate aluminate phases in sulfoaluminate cement can react with SO_4_^2−^ and Cl^−^ ions to form corresponding AFt and chloride-containing AFm phases, respectively [2]. The preparation of CWF involved a water-washing step, which removed part of the soluble chlorides. With increasing CWF replacement, the proportion of high-chloride untreated fly ash in the system decreased, and the characteristic Friedel’s salt peaks at approximately 11.3° and 36.8° gradually weakened. This suggests that the formation of chloride-containing AFm phases may be suppressed.

The variations in AFt and Friedel’s salt in the HW series were generally consistent with those observed in the CW series; however, the characteristic peak of Ca(OH)_2_ was relatively stronger. This may be attributed to the hydrophobic layer formed on fly ash particles, which reduced water wetting and limited the dissolution of active components and soluble salts. Consequently, some hydration reactions were restricted, and the further consumption of Ca(OH)_2_ was reduced. The hydrophobic modification inhibited the rapid release of internal salts and reactive components from fly ash, resulting in a less intensive consumption of Ca(OH)_2_ compared with highly reactive systems. Meanwhile, AFt and chloride-containing AFm phases could still be maintained due to the reduced continuity of free-water pathways, which weakened the migration of aggressive ions. It should be noted that the XRD analysis was mainly used for phase identification and comparison of relative changes among different mixtures. Owing to peak overlap among CaCO_3_, AFt, Friedel’s salt, and carbonate-containing AFm phases, and the absence of Rietveld quantitative analysis, the discussion is limited to characteristic peaks and relative variation trends rather than absolute phase contents.

### 3.6. SEM

**Figure 9:**
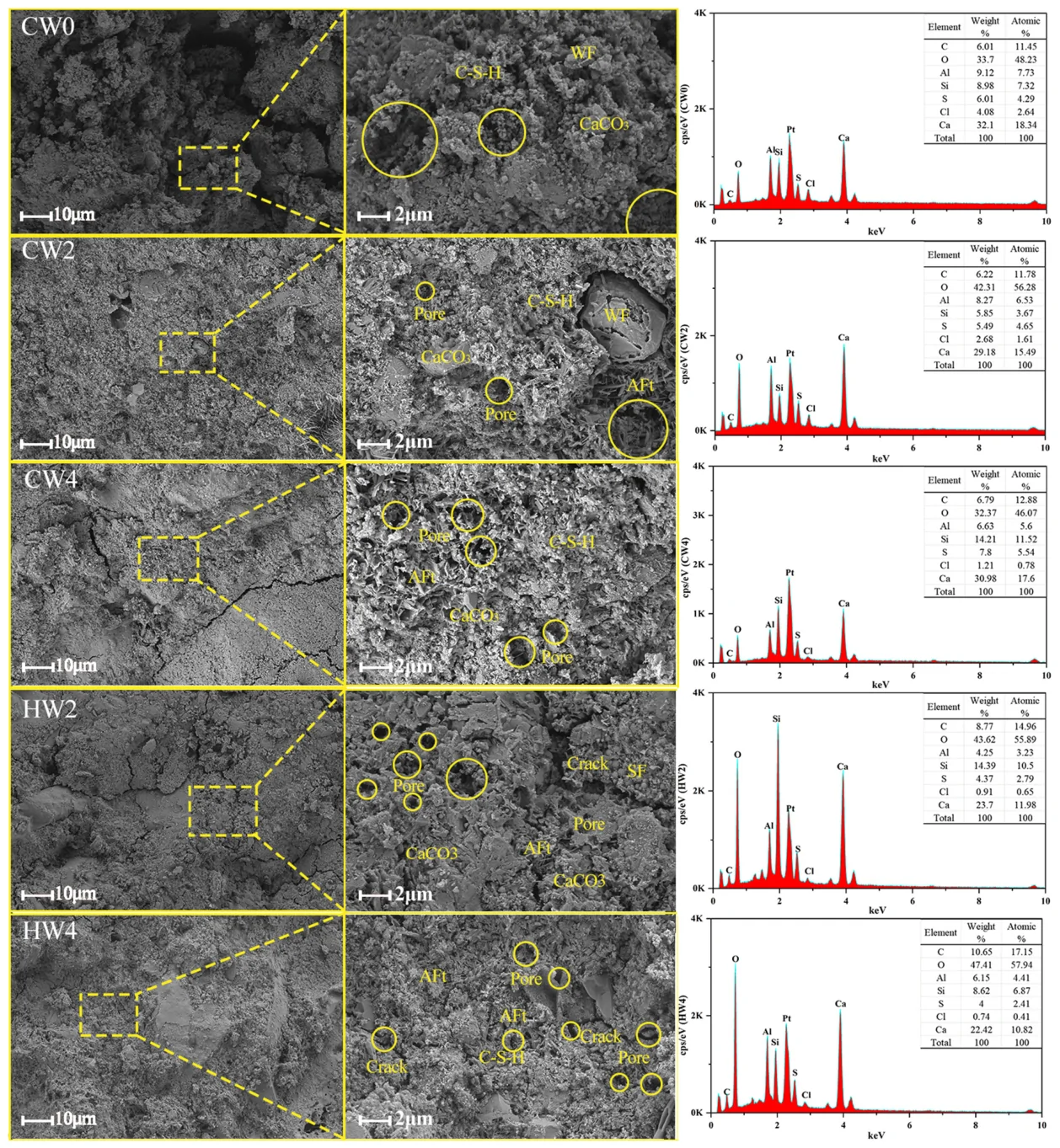
SEM images of mortars with different mix proportions.

The SEM images of mortars with different mix proportions are presented in Figure 9. The CW0 sample exhibited numerous loose agglomerated particles and pores, with some incompletely reacted fly ash particles observed locally. C-S-H gels and a small amount of CaCO_3_ were identified in the matrix, while the hydration products were not sufficiently continuous. EDS analysis showed that the Ca content in CW0 reached 32.10 wt%, and the Cl content was 4.08 wt%, indicating the presence of considerable soluble chlorides or chloride-containing calcium salts in untreated fly ash. It should be noted that EDS results only represent the local semi-quantitative elemental composition of the selected region and cannot directly represent the overall elemental composition of the specimen. These data were used only to assist in comparing variations in local Cl signals among different samples.

Compared with CW0, the matrix structure of CW2 was improved. Fine flocculent and gel-like products were observed between particles, and some unreacted fly ash particles were encapsulated by hydration products. The CaCO_3_ introduced by CWF not only acted as a micro-filler to fill pores but also provided nucleation sites for hydration products, thereby promoting local densification of the matrix [52]. EDS analysis indicated that the Cl content decreased from 4.08 wt% in CW0 to 2.68 wt%. This reduction in the local Cl signal is consistent with the removal of some soluble chlorides during the water-washing step of CWF preparation and the decreased proportion of high-chloride untreated fly ash.

In CW4, abundant C-S-H gels, AFt, and CaCO_3_ phases were observed. CWF promoted carbonate filling and the formation of ettringite in the sulfoaluminate cement system. This morphology was consistent with the enhanced CaCO_3_ diffraction intensity observed in the XRD results. The local semi-quantitative Cl content in the selected region of CW4 further decreased to 1.21 wt%, indicating a decreasing trend in the local Cl signal with increasing CWF replacement.

In the HW2 sample, the hydration products were less continuous than those in the CWF groups. The hydrophobic layer on the fly ash surface weakened the contact between fly ash particles and pore solution, resulting in localized structural defects. This phenomenon may be attributed to the reduced wettability of fly ash particles caused by the hydrophobic coating, which limited the dissolution of reactive components and the continuous growth of hydration products. The local semi-quantitative Cl content in the selected region of HW2 was 0.91 wt%, indicating a relatively weak local Cl signal.

In the HW4 sample, C-S-H and AFt phases were observed, accompanied by a higher number of pores and microcracks. Excessive incorporation of HWF may affect the wetting, dissolution, and continuous growth of hydration products in the cement matrix. The C content increased to 10.65 wt%, indicating an increased proportion of organic hydrophobic groups within the system. The local Cl content in the selected region of HW4 was 0.74 wt%, further indicating a relatively low local Cl signal. In addition, the Ca content decreased to 22.42 wt%, which was lower than that of the CW series samples. This suggests that the hydrophobic layer may restrict the dissolution and reprecipitation of calcium-containing phases and partially inhibit local hydration reactions.

### 3.7. Interfacial Interaction Energy

**Figure 10:**
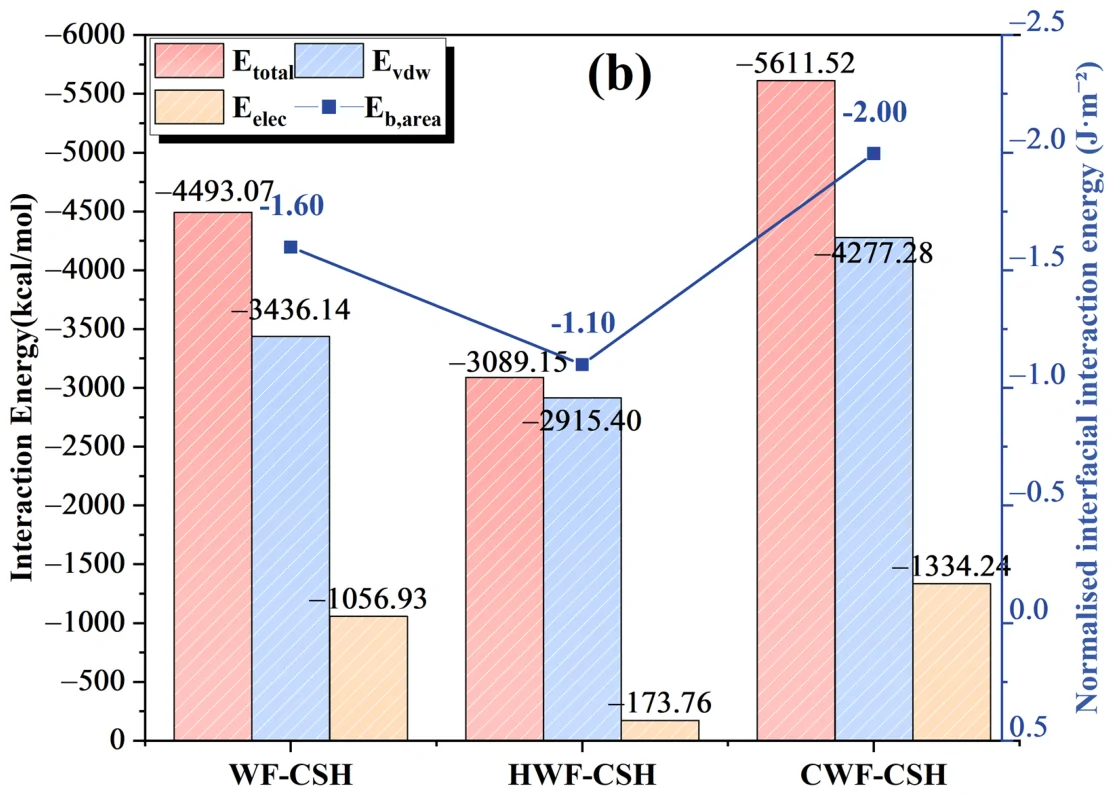
Interfacial interaction energies between three types of fly ash and C-S-H.

The interfacial interaction energies between the three types of fly ash and C-S-H are presented in Figure 10. The total interaction energies of all three systems were negative, indicating the existence of attractive interactions between fly ash and C-S-H interfaces. However, different modification strategies significantly altered the interfacial bonding strength. Among the three systems, the CWF–C-S-H system exhibited the highest absolute interaction energy, with a value of −5611.52 kcal/mol, followed by the WF–C-S-H system (−4493.07 kcal/mol), while the HWF–C-S-H system showed the lowest absolute value (−3089.15 kcal/mol). These results indicate that carbonation modification enhances the interfacial affinity between fly ash and C-S-H, whereas hydrophobic modification significantly weakens their interfacial interactions. The binding energy per unit area exhibited the same trend, with values of −2.00, −1.60, and −1.10 J/m^2^ for CWF–C-S-H, WF–C-S-H, and HWF–C-S-H, respectively. This demonstrates that CWF maintains the strongest interfacial bonding capability even after eliminating the influence of interfacial contact area.

From the perspective of energy components, van der Waals interactions were the dominant contribution to the interfacial bonding of all three systems, with values of −4277.28, −3436.14, and −2915.40 kcal/mol for CWF–C-S-H, WF–C-S-H, and HWF–C-S-H, respectively, accounting for the majority of the total interaction energy. In contrast, electrostatic interactions were more significantly affected by the modification strategies. The electrostatic interaction energy of the CWF–C-S-H system reached −1334.24 kcal/mol, which was higher in magnitude than that of WF–C-S-H (−1056.93 kcal/mol), whereas HWF–C-S-H exhibited the lowest value of only −173.76 kcal/mol. These results indicate that carbonate phases containing calcium and polar active sites generated during carbonation enhanced electrostatic attraction between the fly ash surface and C-S-H, while the low-surface-energy organic layer formed by hydrophobic modification likely shielded polar sites and substantially weakened interfacial electrostatic interactions.

Overall, carbonation modification improved the interfacial stability between fly ash and C-S-H by strengthening van der Waals and electrostatic interactions, whereas hydrophobic modification weakened interfacial bonding due to reduced surface polarity and interfacial affinity. These molecular-scale findings are consistent with the macroscopic experimental results, where CWF promoted hydration and improved mortar strength, while hydrophobic fly ash inhibited hydration and resulted in strength reduction.

### 3.8. Diffusion Behavior and MSD

**Figure 11:**
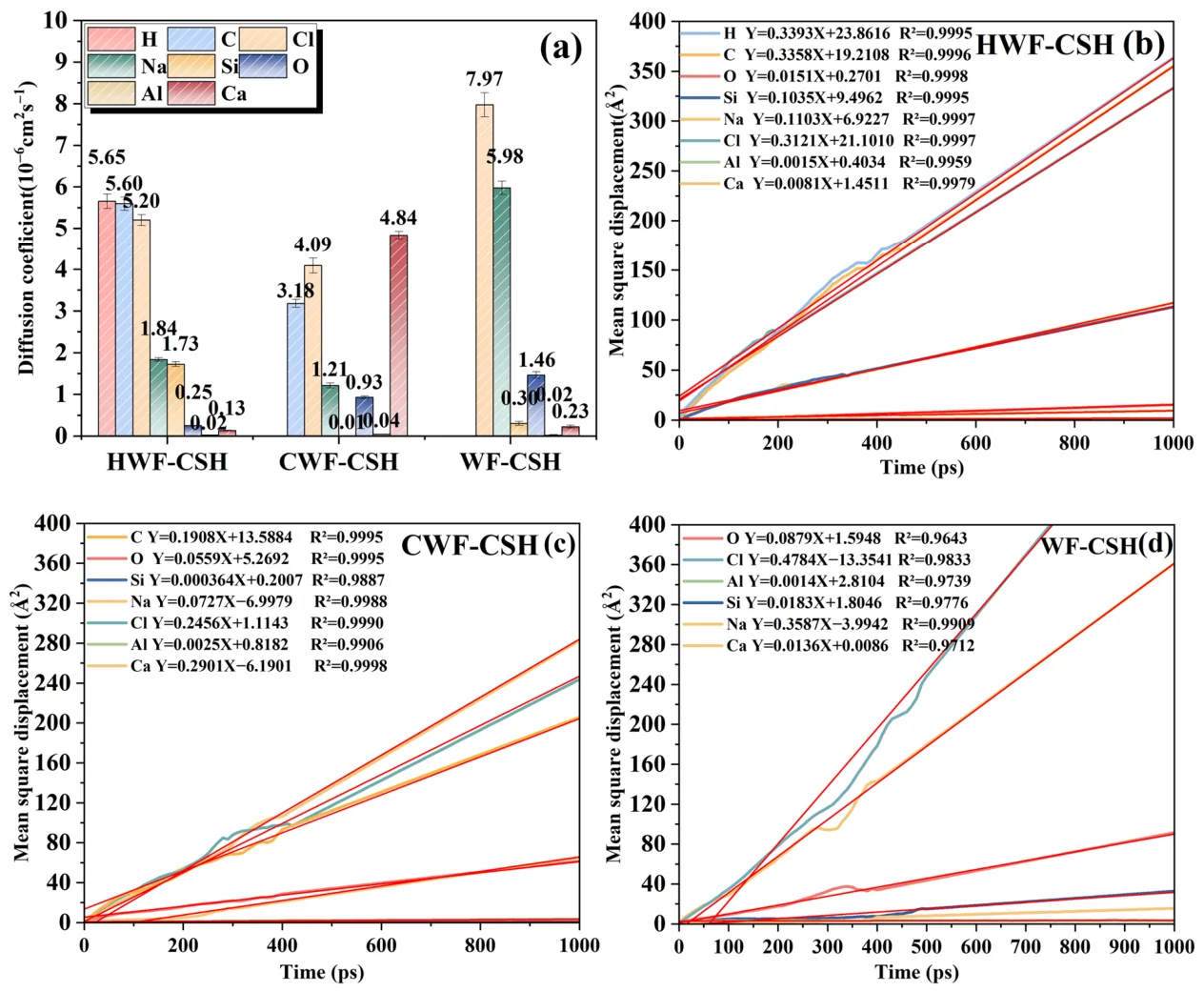
Mean square displacement (MSD) of three types of fly ash components on the C-S-H surface: (**a**) diffusion coefficients of fly ash components on C-S-H; (**b**) HWF–C-S-H; (**c**) CWF–C-S-H; (**d**) WF–C-S-H.

The diffusion coefficients and mean square displacements (MSD) of different components in the three fly ash systems on the C-S-H surface are presented in Figure 11. Figure 11b–d present the corresponding linear fitting equations and coefficients of determination (R^2^). Overall, the major mobile species exhibited good linear relationships within the selected fitting interval, supporting the use of MSD slopes to compare their relative local mobility among the different systems. As shown in Figure 11a, different modification strategies altered the migration behavior of interfacial components in fly ash, with obvious differences observed among the diffusion coefficients of various elements.

The chemical composition of MSWI fly ash is an important factor affecting the local migration behavior of Cl. XRF results showed that the Cl and Na_2_O contents in WF were 9.75% and 8.95%, respectively, higher than those in CWF (7.99% and 6.69%) and HWF (4.12% and 3.17%). This indicates that untreated fly ash contains higher proportions of Cl- and Na-bearing components, consistent with the relatively greater local mobility of Cl and Na in the WF–C-S-H system. For the modified fly ashes, Cl-related behavior is influenced not only by changes in the initial chemical composition, but also by changes in Ca-containing phases induced by water-washing–carbonation treatment and by the altered interfacial chemical environment resulting from hydrophobic modification.

In the untreated WF–C-S-H system, the diffusion coefficients of Cl and Na reached 7.97 × 10^−6^ and 5.98 × 10^−6^ cm^2^·s^−1^, respectively, which were significantly higher than those of other elements. This indicates that soluble chloride salts and alkali metal species in untreated fly ash exhibited relatively strong migration capability at the C-S-H interface. After carbonation and hydrophobic modification, the diffusion coefficient of Cl decreased to different extents. Specifically, the Cl diffusion coefficient was approximately 4.09 × 10^−6^ cm^2^·s^−1^ in the CWF–C-S-H system and 5.20 × 10^−6^ cm^2^·s^−1^ in the HWF–C-S-H system. This indicates that, under the current model conditions, Cl in both modified systems exhibited lower relative local mobility than in the WF–C-S-H system.

Figure 11b shows that the MSD values of H and C in the HWF–C-S-H system continuously increased with simulation time, with diffusion coefficients of 5.65 × 10^−6^ and 5.60 × 10^−6^ cm^2^·s^−1^, respectively, which were relatively high among all components. This behavior was mainly associated with the methylsiloxane organic structures introduced during hydrophobic modification. Compared with inorganic framework elements such as Si, Al, and Ca, organic groups possess greater conformational freedom, enabling more pronounced local motion and conformational rearrangement near the interface. Meanwhile, Na and Cl species still exhibited certain migration capability, whereas the MSD values of Al and Ca remained relatively low throughout the simulation, indicating that hydrophobic modification primarily altered the dynamic behavior of surface organic groups and mobile ionic species while having a limited influence on the stable inorganic framework.

The CWF–C-S-H system exhibited distinct diffusion characteristics compared with the hydrophobic-modified system, as shown in Figure 11c. The MSD values of Cl, C, and some Ca-containing species continuously increased with time, whereas Si and Al maintained relatively low MSD values. This indicates that, after carbonation treatment, mobile species on the fly ash surface could undergo localized rearrangement, while the main inorganic structures, such as Si–O–Al networks, retained relatively high structural stability. Combined with the aforementioned interfacial interaction energy results, the CWF–C-S-H system exhibited stronger interfacial bonding. Therefore, the relatively higher local mobility of these components does not indicate reduced interfacial stability; instead, it likely reflects the redistribution and structural adjustment of calcium- and carbon-containing species under strong interfacial interactions, facilitating the formation of a more stable interface between fly ash particles and C-S-H.

In contrast, the diffusion behavior of the WF–C-S-H system in Figure 11d was mainly dominated by Cl and Na species. With increasing simulation time, the MSD values of these two elements increased significantly, with Cl exhibiting the largest increment, whereas Si, Al, and Ca remained relatively stable. This indicates that, under the current interfacial model conditions, Cl and Na in untreated MSWI fly ash exhibit relatively high local mobility. The higher local mobility of Cl suggests that chloride-containing species in untreated fly ash have greater relative mobility within the present interfacial model. Under the current molecular model conditions, Cl in the CWF–C-S-H and HWF–C-S-H systems exhibits lower relative local mobility than in the WF–C-S-H system. These changes may be associated with the compositional and interfacial structural changes induced by water-washing–carbonation treatment and the altered surface chemical environment resulting from hydrophobic modification, respectively.

## 4. Conclusions

In this study, carbonated and hydrophobically modified MSWI fly ash were directly compared within the same sulfoaluminate cement mortar system. The main conclusions are summarized as follows:

(1) The water-washing–carbonation treatment improved the compatibility of MSWI fly ash with the cementitious matrix and promoted early-age hydration. The compressive strength of the mortar increased by up to 6.8 MPa, while the water absorption decreased by up to 17.8%. Combined XRD, SEM–EDS, and NMR results indicated that the CaCO_3_ formed during carbonation could provide heterogeneous nucleation sites, promote the accumulation of hydration products, and exert a pore-filling effect, thereby enhancing matrix densification. Meanwhile, the water-washing step contributed to the removal of part of the soluble salts. Because no water-washing-only control group was included, the individual contributions of the two treatment steps could not be quantitatively distinguished.

(2) Hydrophobic modification exhibited a trade-off between hydration performance and transport resistance. Although hydrophobic modification delayed hydration and reduced the compressive strength by up to 7.7 MPa, it decreased water absorption by up to 32.5%. SEM observations revealed that hydrophobic groups mainly reduced the wettability of particle surfaces and pore walls, decreased the accessible pore volume for water penetration, and weakened continuous water pathways, rather than promoting structural densification through hydration. Consequently, hydrophobic modification effectively restricted the transport of water and aggressive ions.

(3) Molecular dynamics simulations revealed the interfacial bonding mechanisms between modified fly ash and C-S-H. All three systems exhibited negative interfacial interaction energies, indicating stable attractive interactions between fly ash and C-S-H. Among them, the CWF–C-S-H system showed the strongest interfacial bonding capability, with a binding energy per unit area of −2.00 J/m^2^, which was higher than those of WF–C-S-H (−1.60 J/m^2^) and HWF–C-S-H (−1.10 J/m^2^). The enhanced interfacial interaction of CWF was mainly attributed to strengthened van der Waals and electrostatic interactions, whereas the low-surface-energy organic layer introduced by hydrophobic modification reduced interfacial polarity and weakened interfacial bonding.

(4) MSD analysis showed that, under the current simulation conditions, Cl and Na exhibited relatively high local mobility in the WF–C-S-H system, whereas the local mobility of Cl was reduced in the CWF–C-S-H and HWF–C-S-H systems. These results indicate that different modification methods can alter the dynamic behavior of species at the fly ash–C-S-H interface. The water-washing–carbonation treatment may restrict the local motion of some mobile species by modifying the fly ash composition and strengthening interfacial interactions, whereas hydrophobic modification mainly affects their mobility by altering the surface chemistry and local interfacial environment.

## Figures and Tables

**Table 1:** Chemical compositions of the three types of fly ash.

Chemical Composition	CaO	SiO_2_	Cl	Na_2_O	Al_2_O_3_	SO_3_	P_2_O_5_	Fe_2_O_3_	MgO	K_2_O	TiO_2_	ZnO	CuO	Cr_2_O_3_	Others
WF	33.95	16.78	9.75	8.95	8.25	4.48	4.32	4.1	3.85	2.94	1.41	0.57	0.2	0.06	0.32
CWF	34.99	17.08	7.99	6.69	9.23	4.92	4.12	4.68	3.39	3.45	1.22	0.78	0.23	0.07	1.16
HWF	34.39	28.52	4.12	3.17	8.25	4.35	3.65	4.84	3.14	2.42	1.4	0.81	0.23	0.07	0.64

**Table 2:** Mix proportions of mortars incorporating modified fly ash (kg/m^3^).

Sample	SAC 42.5	P.O 42.5	SF	WF	CWF	HWF	Quartz Sand	Water	SP
CW0	526	222	82	150	0	0	980	294	3
CW1	526	222	82	125	25	0	980	294	3
CW2	526	222	82	100	50	0	980	294	3
CW4	526	222	82	50	100	0	980	294	3
HW1	526	222	82	125	0	25	980	294	3
HW2	526	222	82	100	0	50	980	294	3
HW4	526	222	82	50	0	100	980	294	3

Note: CW0 represents the reference group without modified fly ash. CW and C denote the samples incorporating CWF, whereas HW and H denote the samples incorporating HWF. CW2 represents the sample containing 50 kg/m^3^ of CWF, while HW4 represents the sample containing 100 kg/m^3^ of HWF.

## Data Availability

The original contributions presented in this study are included in the article. Further inquiries can be directed to the corresponding author.
